# Strategies for Updating Social Participation Services: The Case of Baby Boomers in Quebec and Spain

**DOI:** 10.1093/geroni/igaf122.773

**Published:** 2025-12-31

**Authors:** Dolores Majon-Valpuesta, Melanie Levasseur

**Affiliations:** Université de Sherbrooke, Sherbrooke, Quebec, Canada; Université de Sherbrooke Faculty of Medicine and Health Sciences, Sherbrooke, Quebec, Canada

## Abstract

Services designed to promote social participation as a key factor in healthy aging are struggling to meet the expectations of a large generation of older adults, the baby boomer, which in 2021 represented more than a quarter (26%) of the population in both Quebec (Canada) and Spain. Despite the challenge of adapting services to the participation needs of this cohort, little is known about the strategies and changes required to make these services attractive to them. This presentation aims to report results of two qualitative studies exploring the strategies to update services aimed to promote social participation of baby boomer generation. These two studies were both developed in Spain and Quebec with 53 individuals and 12 group interviews, involving a total of 107 baby boomers and 52 community representatives. Three strategies were identified: 1) the consideration of intragenerational diversity from an intersectional perspective, according to criteria such as life trajectories, autonomy levels, identity factors, and daily environment; 2) the organization of services and participation beyond the age criterion, i.e., by interests, needs, common values in which a real interaction between generations is favored; 3) the promotion of services flexible where baby boomers have control over their time, i.e., services friendly to conciliation (caring for oneself, others, continuing professional life, etc.). The services will respond to meaningful participation for members of this generation according to the willingness of decision-makers and service designers to implement these strategies. Enhancing these services leads to greater motivation to participate and responsible use of community resources.

